# Radiographic Aspects of Pentastomiasis in Southern American Bushmaster (*Lachesis rhombeata*)

**DOI:** 10.1111/vru.70040

**Published:** 2025-05-01

**Authors:** Ananda Santiago de Oliveira, Alice Mendes da Silva, Ednilza Maranhão dos Santos, Jozélia Maria de Souza Correia, Fabrício Bezerra de de Sá, Jaqueline Bianque de Oliveira, Fabiano Séllos Costa

**Affiliations:** ^1^ Department of Veterinary Medicine Federal Rural University of Pernambuco Recife Brazil; ^2^ Department of Biology Federal Rural University of Pernambuco Recife Brazil; ^3^ Department of Animal Morphology and Physiology Federal Rural University of Pernambuco Recife Brazil; ^4^ Laboratory of Parasitology Federal Rural University of Pernambuco Recife Brazil

**Keywords:** lung parasites, pentastomida, Radiology, reptiles, snake

## Abstract

Pentastomids are parasites of the respiratory system of reptiles, birds, and mammals, where they can cause lesions resulting in the death of their intermediate hosts. This report describes radiographic aspects of pulmonary pentastomid infection in the Southern American bushmaster (*Lachesis rhombeata*). A female juvenile snake rescued in an urban area of the Northeast region of Brazil presented with lethargic behavior. Radiographic examination of the coelom cavity showed long cylindrical structures in the respiratory system with soft tissue radiodensity and width ranging between 4.0 and 5.0 mm. The next day, the snake died and was submitted to necropsy, where lung parasites were discovered, which were later identified as *Porocephalus stilesi*. A case of correlation between radiographic and macroscopic findings of pentastomid in snakes has not previously been reported.

## 1 Signalment, History, and Clinical Findings

During research carried out in the Imaging diagnostic service of the Veterinary Hospital of the Federal Rural University of Pernambuco, into the radiographic and ultrasonographic anatomy of 15 free‐ranging Southern American bushmasters, we identified that one of the snakes presented with lethargic behavior on clinical examination. This was a female juvenile snake rescued in the urban area of the northeast region of Brazil (8°04’03’’S, 34°55’00’’W) weighing 2.75 kg and with a rostrocloacal length of 125.5 cm. Although there are no hematological reference values for this species, complete blood count showed relative azurophilia and relative heteropenia.

The snake was rescued by federal environmental agencies from the metropolitan region of Recife, Pernambuco state, Brazil. The analysis of this snake was authorized by the Animal Experimentation Ethics Committee of The Federal Rural University of Pernambuco under protocol number 5820080922 and the Biodiversity Authorization and Information System (SISBIO) under protocol number 11218‐1.

## 2 Imaging, Diagnosis, and Outcome

After physical examination, the snake was contained manually using a long transparent acetate tube, without chemical restraint. Segmental radiographs in lateral and dorsoventral views were taken of the entire body, with special attention to the cranial and middle segments for lung evaluation, with overlapping margins, using a computerized radiology system. Radiographic images were obtained by using a mobile X‐ray equipment (Ultra 100, Ecoview, Seoul, Korea) and a direct digitizer (REGIUS SIGMA, Konica Minolta, Tokyo, Japan) placed at a distance of 1 m between the ampule and cassette. Original images were used to analyze the radiographs (WW 4096/WL 2047), and technique settings were 77 Kv and 7 mAs.

**FIGURE 1:**
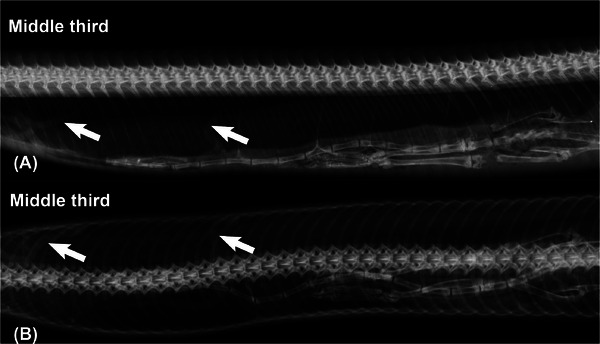
Lateral (A, middle third) and dorsoventral (B, middle third) radiograph of the coelomic cavity of a Southern American bushmaster (*Lachesis rhombeata*). Cylindrical structures in the lung fields are (arrows) compatible with pentastomids. After necropsy, pentastomid infection by *Porocephalus stilesi* was confirmed.

Radiographic examination of the coelom cavity showed long cylindrical structures in the respiratory system with soft tissue radiodensity and width ranging between 4.0 and 5.0 mm and the presumptive diagnosis of pentastomiasis was established. The presumed parasitic elements were positioned in a sinuous manner in the respiratory tract, making it impossible to accurately measure the radiographic length of the parasites (Figure 1).

**FIGURE 2:**
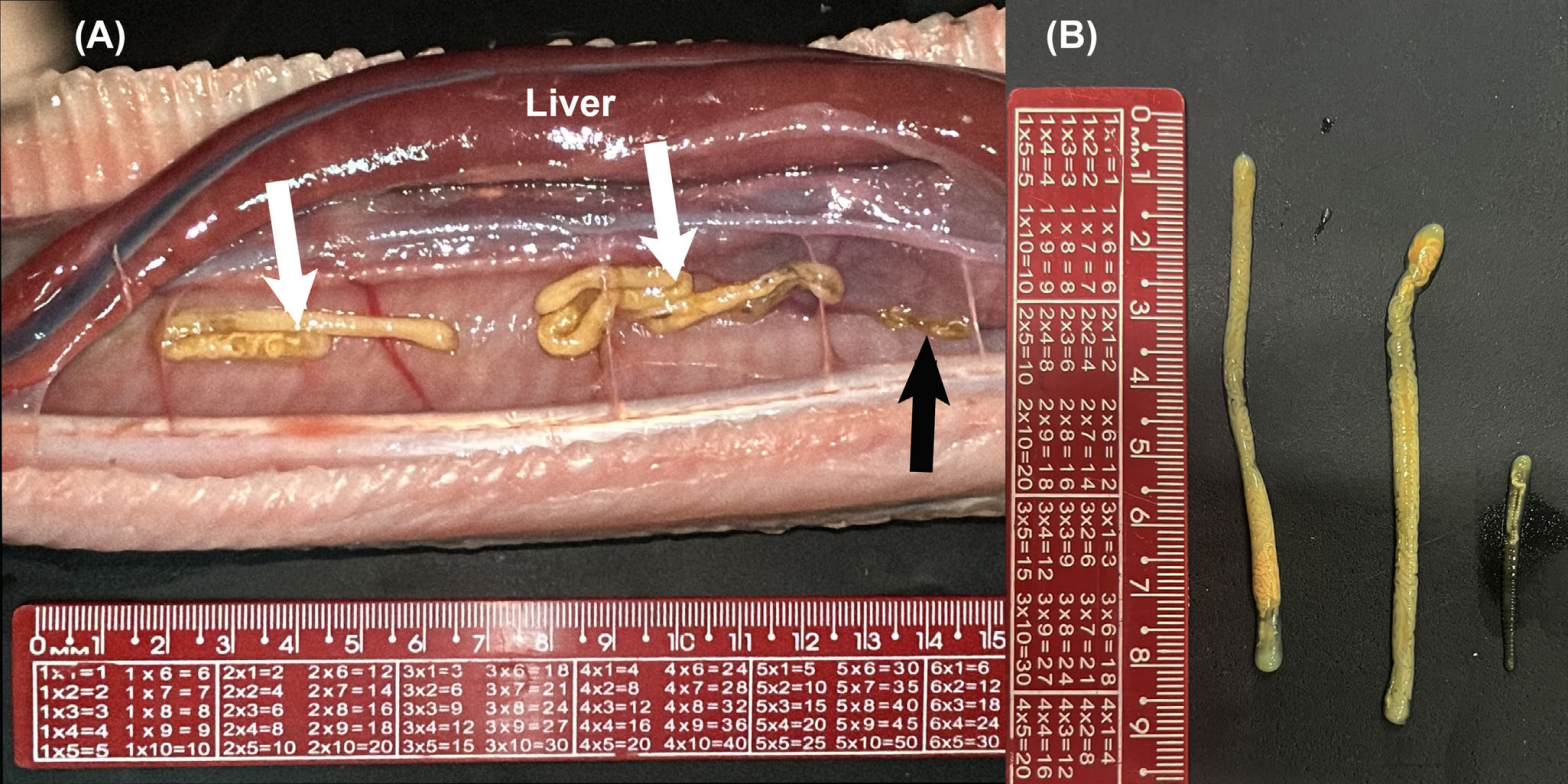
A, B, Necropsy images of a Southern American bushmaster (*Lachesis rhombeata*). The liver was moved to expose the caudal portion of the lung, allowing visualization of the adult pentastosmids measuring approximately 8 cm in length (white arrows). A smaller parasite, possibly male, was also identified (black arrow).

The following day, the snake died, and during the necropsy, three parasites were found in the snake's respiratory system (Figure 2): two larger (7.5 and 7.8 cm) and one smaller (3.1 cm). Microscopic analysis allowed the identification of simple internal hooks and double external hooks in the cephalothorax in the three parasites. In the two larger parasites, the annuli were identified, but in the smaller one, the annuli were not visible. Based on hook characteristics, body length, and annulus number, the parasites, identified as *Porocephalus stilesi*, were two females and one male.

## 3 Discussion

Although radiographic examination has limitations for diagnosing lung diseases in snakes [1, 2], it was able to identify adult female pentastomids in the respiratory tract. The high contrast of the parasite's body with the air within the respiratory system allowed its identification and suggested the degree of parasitic infection through radiographic quantification. Although pentastomids also live in the trachea of snakes [3, 6], no parasites were observed in the trachea of any snake.

For diagnosis, parasitic eggs can be detected through fecal flotation and microscopic examination of sputum, nasal secretions, or lung wash samples [3, 12]. However, the frequency of defecation in snakes is low, making it impractical to conduct routine fecal examinations during a short period under human care. Additionally, due to the absence of clinical signs of respiratory disease, it was not possible to collect nasal secretions for analysis. Respiratory endoscopy was also deemed unfeasible due to the endoscope's incompatibility with the size of the tracheal lumen. Consequently, in this case, radiographic examination showed to be a more viable and noninvasive technique for diagnosing pentastomiasis.

The identification of the pentastomid species was only possible after morphological analysis based on the present simple internal hooks and double external hooks in the cephalothorax, body length (82–91 mm), and annulus number (68–72) in the female parasite and body lengths of the male parasite (56–58 mm) and annuli not visible [15, 16]. The parasite was identified as *P. stilesi*.

Obtaining the radiographs without chemical containment was not a limiting factor for identifying the parasites. With adequate containment care and the use of transparent acetate tubes, the resulting images were diagnostic, as previously reported [1, 2]. Lateral views were more conclusive for the identification and quantification of parasites. Part of the lungs and air sacs are located dorsal to the liver and ventral to the vertebrae within the coelomic cavity [14], and in dorsoventral views, the overlap of these organs reduced radiographic contrast and limited the identification of the parasite. In lateral views, there was no overlap of the lungs with the liver and spine, making identification and counting easier.

Due to the elongated morphological characteristic of snakes, their body is divided into cranial, middle, and caudal thirds to facilitate topographic anatomical description. Segmental radiographs are necessary for a complete assessment of the respiratory tract, with the cranial third housing the trachea and cranial portion of the lung, while the middle third houses the caudal portion of the lung [14].

Male pentastomids have a body thickness of approximately 2.0 mm [8] and in this case report, it was not possible to identify the male parasite in the radiographic images or differentiate it from vascular structures. In this case report, the pentastomids were in the avascular portion of the lung, corresponding to the air sacs, thus excluding the possibility of being confused with pulmonary vasculature. In this report, the radiographic investigation only allowed the identification of adult female pentastomids, and the total parasite load of the snake was underestimated.

No signs of pneumonia were evident in the radiographic images, and at necropsy, the lungs appeared normal. In this report, it was only after necropsy that pneumonia was ruled out since radiographic examination has limitations for snakes, and the diagnosis of lung diseases is late [1, 2, 3], with tomographic examination being the technique of choice [1]. Most pentastomid‐infected snakes present only mild lung disease [3, 4, 8], with mild fibrosis adjacent to the parasite [8] and areas of necrosis [4] being reported histologically. However, more severe secondary pneumonia can occur and may lead to death, especially in captive animals [3, 4]. In general, parasitized animals have poor growth rates, are unhealthy, and are generally more susceptible to disease than parasite‐free animals; thus, effective parasite treatment and prevention are essential [3, 6]. Although ivermectin can be used to treat pentastomiasis [12], there is a risk of mortality because the trachea of snakes is very narrow, often narrower than the width of the pentastomid [14].

**FIGURE 3:**
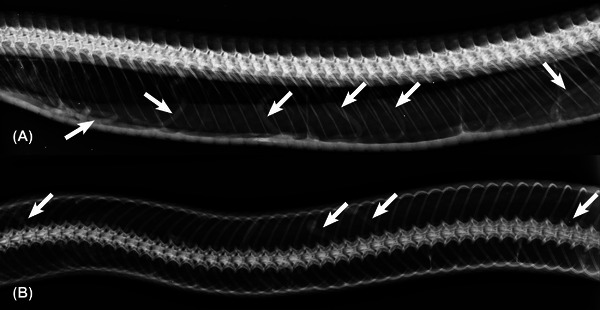
Lateral (A, middle third) and dorsoventral (B, middle third) radiograph of the coelomic cavity of a Southern American bushmaster (*Lachesis rhombeata*). Cylindrical structures in the lung fields (arrows) are compatible with the presumptive diagnosis of pentastomiasis in this case.

Reported cases of infections in reptiles raised as pets reinforce the need for diagnosis and treatment of parasitic infection, when possible [4]. Quarantine is suggested for the introduction of reptiles to avoid disease transmission [3]. Due to the zoonotic potential of pentastomids [8, 9, 10, 11], we suggest that radiographic examinations of the respiratory system of snakes may be an adjunctive diagnostic  for investigating the health of individual animals. In general, free‐ranging snakes present subclinical infections with lower prevalence rates than those found in this study [6, 7]. Recent research demonstrates that pentastomid infections are more prevalent in areas that have suffered human action, which leads to immunosuppression in reptiles [5, 13]. A study with free‐ranging Eastern Indigo snakes (*Drymarchon couperi*) suggested that factors related to physical displacement, habitat modification, and noise pollution cause stress and increase the incidence of pathogens in snakes [5]. Particularly, the Southern American bushmaster snake in this study was rescued from the area of the Atlantic Forest that is being intensely devastated by the expansion of agriculture and urbanization. Although we only have macroscopic and microscopic confirmation of pentastomiasis in one snake, similar radiographic aspects were frequently observed in screening examinations in other bushmasters, suggesting a high prevalence in the region (Figure 3).

In summary, this case reports the diagnosis of pentastomiasis in the *Lachesis rhombeata* by using radiography. The correlation between radiographic and macroscopic findings in the Southern American bushmaster (*L. rhombeata*) may support the diagnosis of this disease in this species or other species of snakes. The use of radiographic images for this purpose is a noninvasive, accessible tool that can contribute to the control of infection in snakes raised as exotic pets, reduce the zoonotic risk of pentastomiasis, as well as assist as a biomarker of possible environmental imbalances in areas degraded by human action.

## List of Author Contributions

### Category 1

(a) Conception and design: Costa
(b) Acquisition of data: Santiago de Oliveira, da Silva, de Sá, Costa
(c) Analysis and interpretation of data: de Oliveira, da Silva, dos Santos, de Sá, Correia, Costa

### Category 2

(a) Drafting the article: Santiago de Oliveira
(b) Revising article for intellectual content: Bianque de Oliveira, dos Santos, de Sá, Correia, Costa

### Category 3

Final approval of the completed article: Bianque de Oliveira, da Silva, dos Santos, de Sá, Correia, Costa

## Conflicts of Interest

The authors declare no conflicts of interest.

## Previous Presentation or Publication Disclosure

The authors have nothing to report.

## Reporting Checklist Disclosure

The authors have nothing to report.

## Data Availability

The data are available from the corresponding author upon reasonable request.
